# SELAdb: A database of exonic variants in a Brazilian population referred to a quaternary medical center in São Paulo

**DOI:** 10.6061/clinics/2020/e1913

**Published:** 2020-08-06

**Authors:** Antonio Marcondes Lerario, Dipika R. Mohan, Luciana Ribeiro Montenegro, Mariana Ferreira de Assis Funari, Mirian Yumie Nishi, Amanda de Moraes Narcizo, Anna Flavia Figueredo Benedetti, Sueli Mieko Oba-Shinjo, Aurélio José Vitorino, Rogério Alexandre Scripnic Xavier dos Santos, Alexander Augusto de Lima Jorge, Luiz Fernando Onuchic, Suely Kazue Nagahashi Marie, Berenice Bilharinho Mendonca

**Affiliations:** I Disciplina de Endocrinologia e Metabologia, Departamento de Clinica Medica, LIM/42, Hospital das Clinicas HCFMUSP, Faculdade de Medicina, Universidade de Sao Paulo, Sao Paulo, SP, BR.; II Department of Internal Medicine, Division of Endocrinology, Metabolism and Diabetes, University of Michigan, Ann Arbor, MI, USA.; III Medical Scientist Training Program, University of Michigan, Ann Arbor, MI, USA.; IV Doctoral Program in Cancer Biology, University of Michigan, Ann Arbor, MI, USA.; V Disciplina de Medicina Molecular, Departamento de Clinica Medica, Faculdade de Medicina FMUSP, Universidade de Sao Paulo, Sao Paulo, SP, BR.; VI Disciplina de Nefrologia, Departamento de Clinica Medica, Faculdade de Medicina FMUSP, Universidade de Sao Paulo, Sao Paulo, SP, BR.; VII Laboratorio de Biologia Molecular e Celular, LIM/15, Departamento de Neurologia, Faculdade de Medicina FMUSP, Universidade de Sao Paulo, Sao Paulo, SP, BR.; VIII Laboratorio de Informatica Medica - LIM/01, Faculdade de Medicina FMUSP, Universidade de Sao Paulo, Sao Paulo, SP, BR.; IX Laboratorio de Sequenciamento em Larga Escala (SELA), Faculdade de Medicina FMUSP, Universidade de Sao Paulo, Sao Paulo, SP, BR.

**Keywords:** Next Generation Sequencing, Database, Mendelian Disorders, Brazil, Population Genetics

## Abstract

**OBJECTIVES:**

High-throughput sequencing of genomes, exomes, and disease-focused gene panels is becoming increasingly common for molecular diagnostics. However, identifying a single clinically relevant pathogenic variant among thousands of genetic polymorphisms is a challenging task. Publicly available genomic databases are useful resources to filter out common genetic variants present in the population and enable the identification of each disease-causing variant. Based on our experience applying these technologies at Hospital das Clínicas da Faculdade de Medicina da Universidade de São Paulo (HCFMUSP), São Paulo, Brazil, we recognized that the Brazilian population is not adequately represented in widely available genomic databases.

**METHODS:**

Here, we took advantage of our 5-year experience as a high-throughput sequencing core facility focused on individuals with putative genetic disorders to build a genomic database that may serve as a more accurate reference for our patient population: SELAdb.

**RESULTS/CONCLUSIONS:**

Currently, our database comprises a final cohort of 523 unrelated individuals, including patients or family members managed by different clinics of HCFMUSP. We compared SELAdb with other publicly available genomic databases and demonstrated that this population is very heterogeneous, largely resembling Latin American individuals of mixed origin, rather than individuals of pure European ancestry. Interestingly, exclusively through SELAdb, we identified a spectrum of known and potentially novel pathogenic variants in genes associated with highly penetrant Mendelian disorders, illustrating that pathogenic variants circulating in the Brazilian population that is treated in our clinics are underrepresented in other population databases. SELAdb is freely available for public consultation at: http://intranet.fm.usp.br/sela

## INTRODUCTION

Advances in high-throughput sequencing technologies in the last decade have enabled systematic genome profiling of thousands of individuals from diverse populations worldwide. Consequently, we now have several publicly available genomic databases that illustrate the profound variability across different ethnic groups, serving as rich resources for investigators seeking to elucidate the molecular basis of human diseases. Indeed, as the cost of next-generation sequencing (NGS) is rapidly declining, NGS-based approaches, including exome sequencing and disease-focused gene panels, are quickly becoming the mainstay of molecular diagnostics.

For example, a clinician who has identified a patient with phenotypic features resembling a particular familial syndrome in the absence of family history may choose to perform exome sequencing using the genomic DNA from this patient, in hopes of identifying a disease-causing genetic variant. However, such an approach routinely results in the identification of thousands of variants. Defining which of these variants is the true disease-causing allele is clinically challenging. An approach that is usually adopted to overcome this obstacle is to filter out common variants in a population. However, even this approach has limited utility, as certain populations are underrepresented in many populational databases, and the clinically relevant allele frequencies remain uncharacterized. At Hospital das Clínicas da Faculdade de Medicina da Universidade de São Paulo (HCFMUSP), São Paulo, Brazil, we adopt NGS-based methods for the diagnosis of putative Mendelian disorders, as a standard practice. However, the most widely utilized databases (e.g. The Genome Aggregation Database (gnomAD) (1), 1000 Genomes (2)) have poor annotation and an underrepresentation of diverse individuals of South American origin (3).

The population of Brazil, in particular, is comprised of many different ethnic groups (3,4), and provides a unique opportunity to identify novel uncharacterized disease-causing variants that are unique to this population. Illustrating this point, an unusually high incidence of pediatric adrenocortical tumors in Brazil (nearly twenty times higher than the global incidence) led to the identification of a novel variant in *TP53* (p.R337H) present in nearly 0.3% of the Southeastern Brazilian population and accounting for 90% of pediatric adrenal tumors in this area (5-7). Subsequent studies demonstrated that the high prevalence of p.R337H in this population can be attributed to a founder effect (5), and highlights the critical need of regionally focused population genetics studies. Recently, investigators at the University of São Paulo Institute of Biology developed a database of exomes from 609 elderly individuals, Arquivo Brasileiro Online de Mutações (ABraOM), which serves as a powerful resource for clinicians and investigators researching this group of individuals (8). Our goal at HCFMUSP was to take advantage of our expertise as an NGS-based facility, with half a decade of experience in using exome sequencing as a diagnostic tool, to create a more representative database of our general patient population.

## MATERIALS AND METHODS

**Subjects.** Our database includes 862 individuals associated with different clinical services (Endocrinology, Neurology, Nephrology, Psychiatric, Gastroenterology, and Rheumatology) at HCFMUSP, comprised of patients with putative Mendelian disorders of uncertain or unknown genetic causes, patients with complex disease traits, patients with sporadic tumors, or unaffected family members. We applied a kinship filter (9) to remove related individuals to produce a final cohort of 523 unrelated Brazilian individuals (240 males, 283 females).

**Sequencing.** Exome sequencing was performed using the Illumina HiSeq 2500 platform in Escola Superior de Agricultura “Luiz de Queiroz” (ESALQ - 2013-2014) or Laboratório de Sequenciamento em Larga Escala da Faculdade de Medicina da Universidade de São Paulo (SELA-FMUSP - 2014-2019). Library preparation and exome capture were performed using the Nextera Rapid Capture Enrichment (Illumina, San Diego, CA) or the SureSelect Target Enrichment System All Exon +/- UTRs V4, V5, and V6 (Agilent Technologies, Santa Clara, CA), according to the manufacturer's instructions.

**Figure 1:**
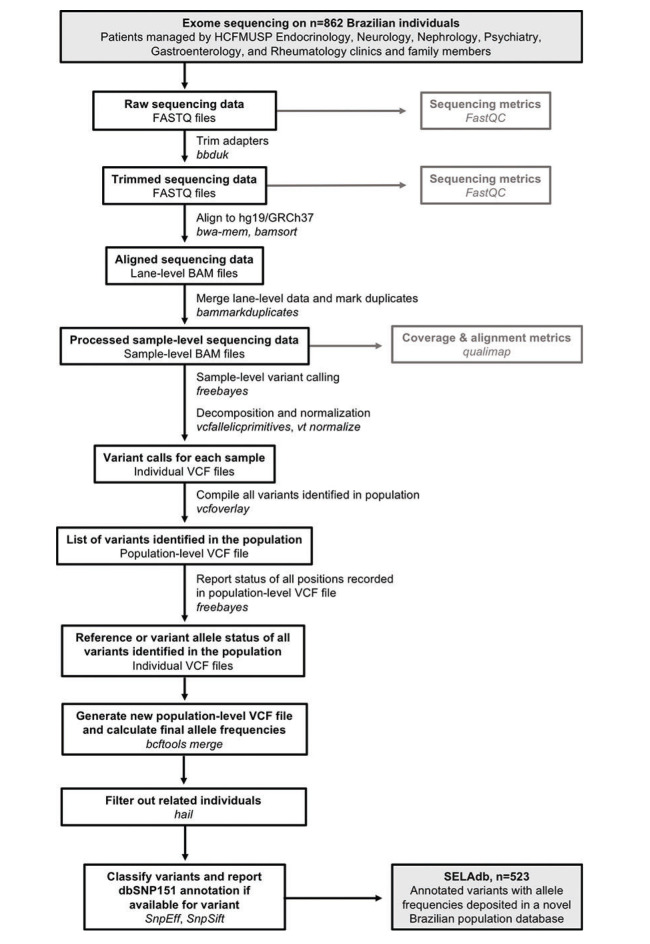
Flow chart of data processing steps used to generate SELAdb database.

**Bioinformatics analysis**. After quality-control using *FastQC*(10) and adapter sequencing removal using the *bbduk* tool from *bbmap* (11), paired-end reads were aligned to the hg19/GRCh37+decoy version of the human genome using *bwa-mem* (12). Aligned reads were then coordinate-sorted, deduplicated, and indexed using *bamsort* and *bammarkduplicate* tools from *biobambam2* (13). Sequencing errors, duplication rates, and coverage metrics were assessed using *qualimap* (14). For variant calling, we used an incremental joint variant calling strategy based on *freebayes* (15), as follows: first, we used *freebayes* to perform variant calling on each bam file, individually. We filtered out low-coverage and low-quality variants (<10x and QUAL<10, respectively). We performed multiallelic sites decomposition, multiallelic variants decomposition, and left normalization of InDels using *vt decompose*, *vcfallelicprimitives* (from *vcflib*), and *vt normalize* tools (16,17). After processing all vcf files, we created a joint list of variants using the *vcfoverlay* (16) tool from *vcflib*. We then used this joint list as an input for *freebayes* (using the -@ option) to perform a second round of variant calling. We used the “-l” switch from *freebayes* to restrict calls only to positions of variants reported in the joint list. This final step enabled us to report all sites from the joint list, even in the samples bearing the reference allele. Sex-specific ploidy was set to regions outside the pseudo-autosomal region of the Y chromosome. Next, we used *bcftools merge* (18) to combine the variant information from individual vcf files into a single file containing the sex-corrected allele frequencies for all the variants detected in the dataset. We then used *hail* (9) to remove related individuals from the dataset. Finally, we used *SNPEff* and *SNPSift*(19,20) to annotate these variants according to the genomic loci, the functional consequences on protein-coding genes, and dbSNP membership. A summary of the bioinformatics workflow employed to construct Sequenciamento em Larga Escala database (SELAdb) is depicted in Figure 1. Finally, we used *somalier* (21) to estimate the ancestry of our population in comparison with the populations included in the 1000 Genomes project, using the individuals in 1000 Genomes as a training set. We represented these ancestry data using principal component analysis (PCA) plots built using *ggplot2* (22) and used a neural network classifier (21) to calculate posterior probabilities for the assignment of a sample in our cohort to the populations defined in 1000 Genomes.

### Ethics

This study was approved by the Institutional Review Board of HCFMUSP, in accordance with the provisions in the Helsinki Declaration. Written informed consent was obtained from all individuals included in SELAdb.

## RESULTS

### SELAdb accurately captures the diversity of the southeastern Brazilian population

As detailed in Figure 1, we analyzed exome sequencing data from 862 individuals to generate a final cohort of exomes from 523 unrelated individuals (240 males, 283 females) and a corresponding list of annotated variants with allele frequencies, which we deposited in a novel Brazilian population database, SELAdb. Among these 523 individuals, we identified 1,788,789 variants, including 1,615,436 single-nucleotide variants, 47,805 small insertions, 121,255 small deletions, and 4,293 complex substitutions (Table 1). Among these variants, 502,738 (28.1%) are not reported in dbSNP151 and, therefore, may represent novel variants present in the Brazilian population (Table 1). A total of 2,973,280 effects (alterations resulting in potential changes in protein structure, function, and/or expression, such as amino acid changes or abrogation of transcription factor binding sites) could be attributed to these 1,788,789 variants, including 581,757 targeting annotated exons. Included among these are 152,984 synonymous, 207,854 missense, 3,742 stop-gained, 256 stop-lost, 405 start-lost, and 9,653 frameshift variants (Table 2).

**Figure 2:**
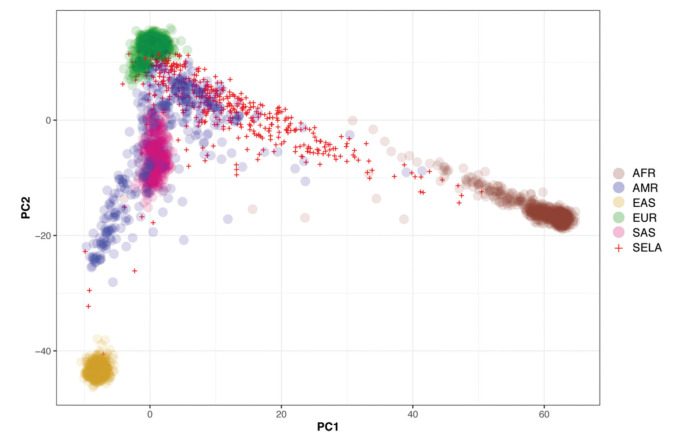
Biplot depicting the two principal components (x-axis: PC1, y-axis: PC2) capturing the highest genetic variation among different populations from 1000 Genomes (colored circles) and SELAdb individuals (red crosses). Each circle or cross represents a single individual. The populations represented by the colored circles are African (AFR), Ad-Mixed American (AMR), East Asian (EAS), European (EUR), and South Asian (SAS).

**Figure 3:**
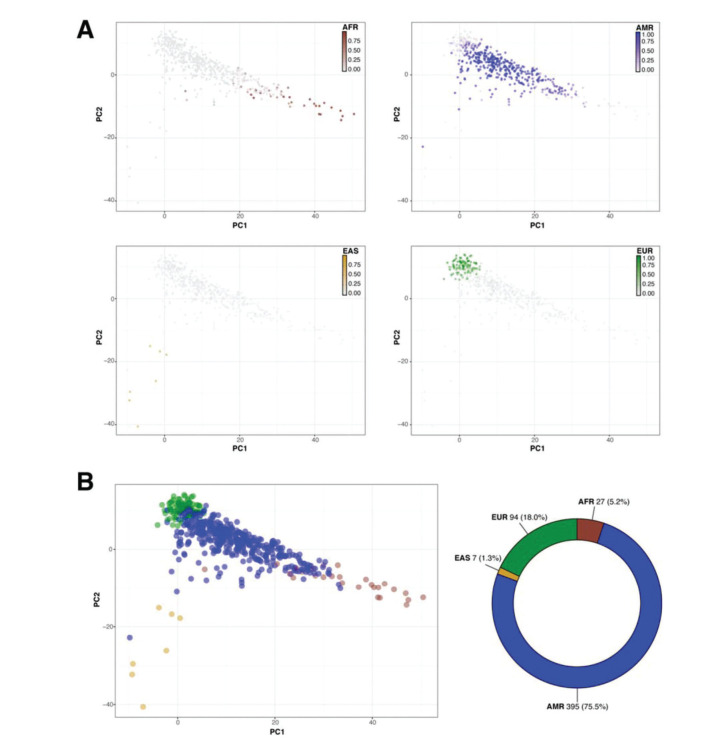
Classification of SELAdb individuals using a neural network classifier and 1000 Genomes populations as the training set. Panel A depicts a series of biplots (x-axis: PC1, y-axis: PC2) representing the PCA analysis of SELAdb individuals, which are color-coded according to the posterior probabilities of being classified as a distinct population by using 1000 Genomes, including AFR (brown, upper left), AMR (blue, upper right), EAS (yellow, lower left), and EUR (green, lower right). Panel B depicts a biplot (left; x-axis: PC1, y-axis: PC2) representing each SELAdb individual, which is color-coded according the final classification given by the neural network classifier. The final distribution of individuals in each category are represented in the donut plot (right). Consistent with the distribution of SELAdb individuals in Panel A, most SELAdb individuals are classified as AMR.

Then, we sought to determine the ancestry of the individuals in our cohort using the populations defined by the 1000 Genomes project as a reference. The biplot shown in Figure 2 represents the first two principal components that capture most of the variability present in the data. The European (EUR), African (AFR), and East Asian (EAS) populations encompass three well-defined clusters in the extremes of this two-dimensional space, forming the vertices of a triangle. As expected, the admixed American population (AMR) exhibits higher variability and is dispersed over a larger area of the plot, with limbs extending to the areas delimited by the EUR, AFR, and EAS populations (Figure 2). Similar to previous studies, our results indicate that SELAdb, comprising urban southeastern Brazilian individuals, forms a continuum between EUR and AFR populations, which is consistent with a high degree of intermarriage between these two populations in southeastern Brazil (3). Furthermore, we observe here that individuals in SELAdb broadly overlap with a large fraction of the AMR population (Figure 2). To better quantify and define the putative ancestry of individuals in SELAdb, we applied a neural network classifier to fit individuals in SELAdb to pre-existing EAS, SAS, EUR, AFR, and AMR categories in 1000 Genomes (Figure 3). This analysis revealed that the majority of individuals in SELAdb could, indeed, be classified as AMR (75.5%), followed by EUR (18.0%), AFR (5.2%), and EAS (1.3%) (Figure 3B).

**Figure 4:**
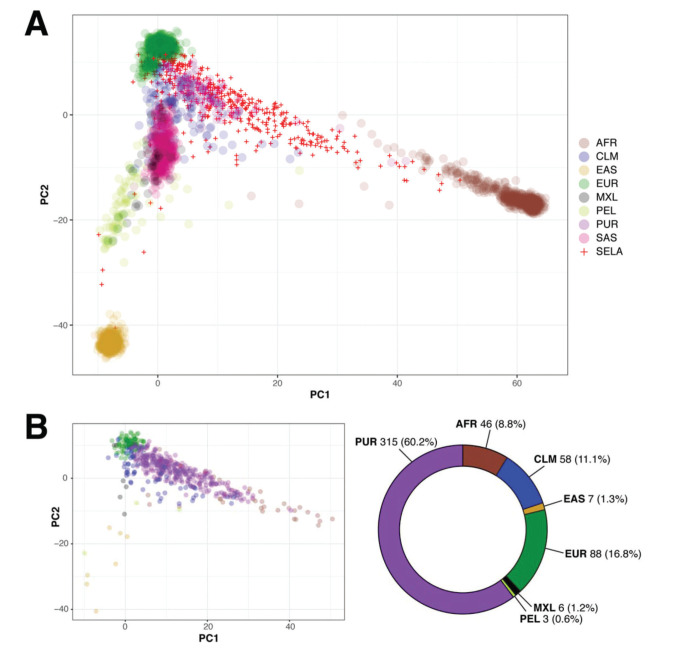
Panel A: biplot (x-axis: PC1, y-axis: PC2) showing the overlap between 1000 Genomes (colored circles) and SELAdb individuals (red crosses). In this representation, the AMR population is split according to its subpopulations, including Colombians (CLM), Mexicans of Los Angeles (MXL), Peruvians of Lima (PEL), and Puerto Ricans (PUR). Panel B: Neural network classification of SELAdb individuals using the 1000 Genomes as a training set. Consistent with the distribution of SELAdb individuals in Panel A, most SELAdb individuals classify as PUR.

The AMR population in 1000 Genomes is a heterogeneous group comprised of individuals from different geographic regions of Latin America, but does not include any Brazilian individuals (2). Given that the majority of individuals in SELAdb is classified as AMR, despite the absence of Brazilian individuals in this set, we sought to determine which subgroup of the AMR population in 1000 Genomes is the most similar to individuals from SELAdb. To address this question, we performed a similar PCA analysis, but this time with the AMR population separated into its constituent subpopulations, including Peruvians of Lima (PEL), Mexicans of Los Angeles (MXL), Colombians (CLM), and Puerto Ricans (PUR) (Figure 4A). From this analysis, we can appreciate that the AMR population is comprised of unique subclusters, in which PEL, MXL, CLM, and PUR populations cluster separately within the region spanned by AMR in Figure 2. This observation suggests that the broad space encompassed by AMR in the biplot may be partially explained by the number of populations of diverse origins defined as AMR, rather than by a broad heterogeneity dispersed throughout each population. Indeed, Figure 4A illustrates that the MXL and PEL form a distinct cluster that localizes closer to EAS, and farther from EUR and AFR; this observation is consistent with the theory of the Asian origin of native American populations (23). In contrast, PUR exhibit a distinct pattern, characterized by a higher influence of both EUR and AFR. Finally, CLM exhibit characteristics of both MXL/PEL and PUR. We observe here that SELAdb largely overlaps with PUR, which is consistent with the stronger influence of EUR and AFR in the southeastern Brazilian population (3). By further quantifying this overlap using a neural network classifier and the subdivided AMR groups (Figure 4B), we could observe that the majority of individuals in SELAdb are classified as PUR (60.2%). This observation suggests a similar contribution of European and African ancestries to both PUR and the southeastern Brazilian populations, which is consistent with common historical aspects of colonization of both geographical regions and recent studies defining the ancestral contributions to each population (3,24).

### SELAdb enables identification of novel, potentially disease-causing variants in a Brazilian population

Given the ability of SELAdb to identify novel variants present in the Brazilian population, that are distinct from other population databases (Table 1), we sought to evaluate its utility in identifying well documented pathogenic variants in a set of 60 genes associated with highly penetrant genetic disorders according to the American College of Medical Genetics and Genomics (ACMG SF v2.0) (25). We were particularly interested in this set of genes, as variants are related with a high risk of diseases associated with early mortality, including cardiovascular disease and familial neoplasia syndromes. Therefore, we suspected that many of these variants may be unique to the Brazilian population but absent from ABraOM database, given its inclusion criteria (8).

We identified 24 known pathogenic or likely pathogenic variants according to ClinVar (26) (Table 3). Interestingly, 11 of these variants are exclusive to SELAdb, not being reported in gnomAD and ABraOM. Thirteen variants were also reported in gnomAD and/or ABraOM (7 in gnomAD and ABraOM, 6 in gnomAD only, 0 in ABraOM only). In addition, we identified 7 variants that were predicted to be pathogenic, according to InterVar (27), among which only 4 were present in gnomAD and/or ABraOM (Table 4). These observations highlight the unique contribution of SELAdb in augmenting the spectrum of potential disease-causing variants present in the Brazilian population and illustrate its clinical and research utility.

## DISCUSSION

The Brazilian population is highly admixed, being comprised of several different ethnic groups, and inadequately represented in publicly available genomic databases (3,4). Here, we took advantage of our 5-year experience as a large-scale sequencing core facility to build a representative local genomic database for the southeastern Brazilian population, SELAdb. Although many individuals included in SELAdb are patients or family members with rare Mendelian disorders, contributing to the identification of novel disease-causing variants (28-40), our analyses demonstrate that it adequately represents our local patient population. Through ancestry analysis, we observed that the population captured by SELAdb bears diverse genetic influences, which are characteristic of the admixed southeastern Brazilian population, similar to previous reports (3). In this analysis, we also identified a large overlap between SELAdb individuals and the 1000 Genomes AMR population (Figures 2-3), especially regarding PUR individuals (Figure 4). Taken together, our analyses illustrate the importance of regional population databases in better representing individuals of diverse origin.

Our effort adds value to another recently launched genetic database on Brazilian individuals, ABraOM (8). However, given the focus of ABraOM on healthy elderly individuals, pathogenic variants that are present in our patient population may be underrepresented. In contrast, our database is focused on a population of patients or family members with putative genetic diseases in whom we have identified a spectrum of known and potentially novel pathogenic variants, as illustrated in Tables 3 and 4. We believe that SELAdb may be informative regarding the prevalence of pathogenic variants in the southeastern Brazilian population and facilitate future genetics studies on Brazilian individuals.

In conclusion, SELAdb is a publicly available database that is representative of our regional patient population. We believe that, in addition to ABraOM, SELAdb will be a valuable resource for investigators using genomics data from the Brazilian population. SELAdb is rapidly increasing in size; updates and improvements, including more detailed phenotypic annotations associated with specific variants, are expected to be implemented every six months. The data can be freely accessed at http://intranet.fm.usp.br/sela.

## AUTHOR CONTRIBUTIONS

Lerario AM was responsible for the conceptualization, data analysis, manuscript writing and supervision. Mohan DR was responsible for the data analysis and manuscript writing. Benedetti AFF was responsible for the data analysis and website construction. Montenegro LR, Funari MFA, Nishi MY, Narcizo AM, Oba-Shinjo SM were responsible for the data generation. Vitorino AJ, Santos RASX were responsible for the website and database construction. Jorge AAL, Onuchic LF, Marie SKN and Mendonça BB were responsible for the project coordination, supervision and manuscript editing.

## Figures and Tables

**Table 1:** SELAdb variants classified by type and presence in other databases.

**Total number of SELAdb variants**	**1,788,789**
**Variant type**	**Number of variants (% of SELAdb)**
Single nucleotide polymorphism (SNP)	1,615,436 (90.3%)
Insertion	47,805 (2.7%)
Deletion	121,255 (6.8%)
Mixed	4,293 (0.2%)
**Database**	**Number of variants annotated (% of SELAdb)** **Number of novel variants (% of SELAdb)**
dbSNP151	*Annotated* - 1,286,051 (71.9%) *Novel* - 502,738 (28.1%)
1000 Genomes	*Annotated* - 1,205,879 (67.4%) *Novel* - 582,910 (32.6%)
gnomAD	*Annotated* - 489,563 (27.4%) *Novel* - 1,299,226 (72.6%)
ABraOM	*Annotated* - 915,702 (51.2%) *Novel* - 873,087 (48.8%)

**Table 2:** Number of effects attributed to variants in SELAdb by region and type.

Region	Count	Percentage (%)
Downstream	402,130	13.525
Exon	581,757	19.566
Gene	16	0.001
Intergenic	60,291	2.028
Intron	1,091,611	36.714
Motif	8,440	0.284
Splice site acceptor	1,387	0.047
Splice site donor	1,604	0.054
Splice site region	35,011	1.178
Transcript	199,903	6.723
Upstream	374,792	12.605
UTR 3 prime	165,649	5.571
UTR 5 prime	50,689	1.705
**Type**	**Count**	**Percentage (%)**
3’ UTR variant	165,652	5.941
5’ UTR premature start codon gain variant	6,053	0.201
5’ UTR truncation	3	0
5’ UTR variant	44,637	1.48
TFBS ablation	75	0.002
TFBS variant	8,365	0.277
Bidirectional gene fusion	7	0
Conservative inframe DEL	1,150	0.038
Conservative inframe INS	480	0.016
Disruptive inframe DEL	2,133	0.071
Disruptive inframe INS	655	0.022
Downstream gene variant	402,132	13.331
Exon loss variant	3	0
Frameshift variant	9,653	0.32
Gene Fusion	9	0
Initiator codon variant	38	0.001
Intergenic variant	60,291	1.999
Intragenic variant	174,620	5.789
Intron variant	1,123,430	37.241
Missense variant	207,854	6.89
Noncoding transcript exon variant	164,421	5.45
Noncoding transcript variant	90	0.003
Protein-protein contact	476	0.016
Sequence feature	25,193	0.835
Splice acceptor variant	1,414	0.047
Splice donor variant	1,675	0.056
Splice region variant	40,619	1.347
Start lost	405	0.013
Stop gained	3,742	0.124
Stop lost	256	0.008
Stop retained variant	123	0.004
Structural interaction variant	43,197	1.432
Synonymous variant	152,984	5.071
Upstream gene variant	374,792	12.424

UTR=untranslated region, TFBS=transcription factor binding site, DEL=deletion, INS=insertion.

**Table 3:** SELAdb variants classified as pathogenic/likely pathogenic using ClinVar and recommended to be reported by ACMG.

Gene	Mutation	dbSNP151	SELAdb allele frequency	Other databases
*MUTYH*	NM_001128425:p.479_480del	rs587778541	1/1042	gnomAD, ABraOM
*MUTYH*	NM_001128425:p.Gly396Asp	rs36053993	6/1044	gnomAD, ABraOM
*PCSK9*	NM_174936:p.Tyr142X	rs67608943	1/1046	gnomAD, ABraOM
*LMNA*	NM_001282625:p.Cys522X	N/A	1/1044	SELAdb only
*TNNT2*	NM_001001430:p.Asn271Ile	N/A	1/1046	SELAdb only
*PKP2*	NM_004572:c.2578-2A>C	N/A	1/1016	SELAdb only
*BRCA2*	NM_000059:p.Tyr2154fs	rs80359596	1/970	SELAdb only
*ATP7B*	NM_000053:exon17:c.A3694C:p.T1232P	rs568009639	1/1046	gnomAD, ABraOM
*ATPB7*	NM_000053:p.Pro1134fs	rs137853281	2/1044	gnomAD, ABraOM
*ATPB7*	NM_000053:p.His1069Gln	rs76151636	1/1046	gnomAD
*ATPB7*	NM_000053:p.Pro840Leu	rs768671894	1/1046	SELAdb only
*ATPB7*	NM_000053:p.Leu708Pro	rs121908000	1/1034	SELAdb only
*BRCA1*	NM_007294:p.Cys903X	N/A	2/1030	SELAdb only
*BRCA1*	NM_007294:p.Cys64Arg	rs80357064	1/1004	SELAdb only
*LDLR*	NM_000527:p.Gly55Gly	rs150644181	1/1046	gnomAD, ABraOM
*LDLR*	NM_000527:p.Glu418Lys	N/A	1/1038	gnomAD
*LDLR*	NM_000527:p.Gly592Glu	rs137929307	1/1038	gnomAD
*LDLR*	NM_000527:p.Arg744X	rs200793488	1/1046	SELAdb only
*TNNI3*	NM_000363:p.Asp196Asn	rs104894727	1/1042	gnomAD
*TNNI3*	NM_000363:p.Arg145Gln	rs397516349	1/1026	gnomAD
*APOB*	NM_000384:p.Ala13fs	N/A	2/1008	SELAdb only
*APOB*	NM_000384:p.F2181fs	N/A	1/916	gnomAD
*PMS2*	NM_000535:c.989-2A>G	rs587779347	1/890	SELAdb only
*PMS2*	NM_000535:p.Met1Val	rs587779333	1/1032	gnomAD, ABraOM

NOTE. Regarding the *BRCA1* mutation NM_007294:p.Cys903X, with allele frequency 2/1030: both alleles are present in the same individual in SELAdb. The phenotype of this individual is described in Freire et al. Eur. J. Med. Genet. 2018 (PMID: 29133208).

**Table 4:** SELAdb variants that were classified as pathogenic using InterVar and recommended to be reported by ACMG.

Gene	Mutation	dbSNP151	SELAdb allele frequency	Other databases
*PCSK9*	NM_174936:p.Gln387X	N/A	1/1020	SELAdb only
*CACNA1S*	NM_000069:p.Arg1702X	rs550371466	1/1044	gnomAD
*RYR2*	NM_001035:p.Cys1914X	N/A	1/1030	SELAdb only
*SCN5A*	NM_198056:p.Glu1053Lys	rs137854617	1/1010	gnomAD, ABraOM
*RET*	NM_020630:p.Val804Met	rs79658334	1/934	gnomAD, ABraOM
*MYBPC3*	NM_000256:p.Gly278X	N/A	1/978	SELAdb only
*ATP7B*	NM_000053:p.Gly170X	N/A	1/1022	gnomAD
